# Dr. Clinton Cushing

**Published:** 1904-06

**Authors:** 


Dr. Clinton Cushing, of San Francisco, died at his winter home at Washington, D. C., May 10, 1904, aged 63 years. He graduated from Rush Medical College in 1865, and removed to California during the same year. He served as a medical officer in the United States navy in the early 60's; later he became professor of gynecology in the Cooper Medical College and consulting surgeon to the French Hospital. He was a founder of the American Association of Obstetricians and Gynecologists, but resigned in 1900, on account of ill health. During his active career he was one of the most prominent abdominal surgeons on the Pacific coast.

